# Transfer Function Analysis of Respiratory and Cardiac Pulsations in Human Brain Observed on Dynamic Magnetic Resonance Images

**DOI:** 10.1155/2013/157040

**Published:** 2013-04-24

**Authors:** Yi-Hsuan Kao, Wan-Yuo Guo, Adrain Jy-Kang Liou, Ting-Yi Chen, Chau-Chiun Huang, Chih-Che Chou, Jiing-Feng Lirng

**Affiliations:** ^1^Department of Biomedical Imaging and Radiological Sciences, National Yang-Ming University, Taipei 112, Taiwan; ^2^School of Medicine, National Yang-Ming University, Taipei 112, Taiwan; ^3^Department of Radiology, Taipei Veterans General Hospital, Taipei 112, Taiwan; ^4^Laboratory of Integrated Brain Research, Department of Medical Research and Education, Taipei Veterans General Hospital, Taipei 112, Taiwan

## Abstract

Magnetic resonance (MR) imaging provides a noninvasive, *in vivo* imaging technique for studying respiratory and cardiac pulsations in human brains, because these pulsations can be recorded as flow-related enhancement on dynamic MR images. By applying independent component analysis to dynamic MR images, respiratory and cardiac pulsations were observed. Using the signal-time curves of these pulsations as reference functions, the magnitude and phase of the transfer function were calculated on a pixel-by-pixel basis. The calculated magnitude and phase represented the amplitude change and temporal delay at each pixel as compared with the reference functions. In the transfer function analysis, near constant phases were found at the respiratory and cardiac frequency bands, indicating the existence of phase delay relative to the reference functions. In analyzing the dynamic MR images using the transfer function analysis, we found the following: (1) a good delineation of temporal delay of these pulsations can be achieved; (2) respiratory pulsation exists in the ventricular and cortical cerebrospinal fluid; (3) cardiac pulsation exists in the ventricular cerebrospinal fluid and intracranial vessels; and (4) a 180-degree phase delay or inverted amplitude is observed on phase images.

## 1. Introduction 

 Human brain mostly is comprised of brain tissues, blood, and cerebrospinal fluid (CSF). These components maintain a fixed volume, while they are protected by and confined in the skull. According to the Monro-Kellie doctrine, in a fixed volume an increase in the volume of one cranial component must be compensated by the decrease in volume of other cranial components [1]. The modulations of intracranial pressure by respiratory and cardiac pulsations are observed by using lumbar, cisternal, and ventricular puncture [2]. Magnetic resonance (MR) imaging, however, provides a noninvasive, *in vivo* imaging technique for studying respiratory and cardiac pulsations in human brains [3–9], because these pulsations can be recorded as flow-related enhancement on dynamic MR images. 

 Independent component (IC) analysis is a blind source separation technique [10]. It is described as a partial volume calculation technique when applied to analyze dynamic MR images [11]. The outputs of IC analysis are IC images and corresponding signal-time curves. The output IC images provide a coarse segmentation for voxels with different signal-time curves, and the IC images are assumed to be spatially independent. It has been used to analyze functional MR images [12–14] and to detect cluster microcalcification breast cancer [15]. By applying IC analysis to dynamic MR images, respiratory and cardiac pulsations are observed at intracranial arteries and CSF [16]. However, the propagation of respiratory and cardiac pulsations in the brain is not yet clear and needs further investigation.

The transfer function analysis is used to study the relationship between input and output signals of a linear time-invariant system [17]. The magnitude and phase of a transfer function reflects the amplitude change and temporal delay from input signals to output signals at different frequency bands. The transfer function between arterial blood pressure and cerebral blood flow velocity in the middle cerebral arteries is used to study cerebral autoregulation in normal subjects [18], in patients with occlusive cerebrovascular diseases and arteriovenous malformations [19], and in patients with carotid stenosis [20]. In functional MR imaging research, transfer function provides information on the temporal delays between regions for investigating how these regions within a network interact with each other [21]. In this study, we applied transfer function analysis to dynamic MR images to investigate the respiratory and cardiac pulsations in the brain of normal subjects.

## 2. Materials and Methods

### 2.1. Data Acquisition

Dynamic MR images were acquired from normal subjects on a 1.5-Tesla MR scanner (Signa CV/i, GE Medical Systems, Milwaukee, WI, USA). Written informed consent was obtained. A single-shot, gradient-echo, echo-planar imaging pulse sequence was used for acquiring the images. The scan parameters were *T*_*E*_/*T*_*R*_ = 60/200 ms, flip angle = 90°, field of view = 24 × 24 cm, image matrix = 128 × 128, slice thickness = 5 mm, and one signal averaging. The wavelength of the crusher gradient was increased from 1 ms to 10 ms for suppressing residual transverse magnetic dipole moment [22]. Five hundred and twelve dynamic images were acquired from transaxial planes through and above the third ventricle. The subjects were instructed to breathe normally during the scanning. Image postprocessing procedure was done offline on a personal computer by using software programs written in MATLAB (MathWorks, Inc., Natick, MA, USA).

### 2.2. Periodogram

The frequencies of respiratory and cardiac pulsations measured from a subject were not stationary during the scan. We calculated a periodogram [23] from the acquired images for choosing an appropriate segment of the data for postprocessing procedure. The 512 dynamic images were arranged into 213 segments with each segment containing 300 sequential dynamic images. Within each segment, the amplitude spectra for the 300 dynamic images were calculated on a pixel-by-pixel basis by using discrete Fourier transform. The sum of the amplitude spectra of all pixels was used to represent this segment. A two-dimensional periodogram was generated, in which the horizontal axis was the starting image number, in which the vertical axis was the frequency, and the gray level was used to represent the amplitude spectra. A segment with most stationary respiratory and cardiac pulsations in the periodogram was selected for the following postprocessing procedure.

### 2.3. Independent Component Analysis

Temporal mean and standard deviation images were calculated from the selected 300 dynamic images. The temporal mean was subtracted from the dynamic images to produce zero-temporal-mean dynamic images. The FastICA technique [10] was applied to the zero-temporal-mean dynamic images for finding respiratory and cardiac pulsations. We used four output ICs in this experiment.

### 2.4. Transfer Function Analysis

For a linear time-invariant system, the output signal, *y*(*t*), can be expressed as a convolution of the input signal, *x*(*t*), and the system response function, *h*(*t*), described by

(1) $$\begin{array}{c} y\left(t\right)=x\left(t\right)⊗h\left(t\right), \end{array}$$

where ⊗ indicates a convolution calculation [17]. The previous equation also can be expressed in the frequency domain as

(2) $$\begin{array}{c} Y\left(f\right)=X\left(f\right)H\left(f\right), \end{array}$$

where *X*(*f*), *Y*(*f*), and *H*(*f*) are the Fourier transform of *x*(*t*), *y*(*t*), and *h*(*t*), respectively. The transfer function, *H*(*f*), is calculated as

(3) $$\begin{array}{c} H\left(f\right)=\left|H\left(f\right)\right|\exp\left\{i\phi \left(f\right)\right\}=\frac{Y\left(f\right)}{X\left(f\right)}, \end{array}$$

where |*H*(*f*)| and *ϕ*(*f*) are the magnitude and phase of the transfer function at frequency *f*, respectively. The magnitude and phase of *H*(*f*) reflect the amplitude change and temporal delay from *x*(*t*) to *y*(*t*) at different frequencies, respectively. A phase delay can be calculated as the following equation:

(4) $$\begin{array}{c} \tau_{p}\left(f\right)=-\frac{\phi \left(f\right)}{2\pi f}. \end{array}$$

The *τ*_*p*_(*f*) value provides information on the temporal delay of the output signal, *y*(*t*), in a periodical waveform at this specific frequency, as compared with the input signal, *x*(*t*).

For a global analysis, the transfer function analysis was applied to the four complex-valued output spectra of the FastICA calculation results, by using one spectrum as *X*(*f*) and the other three spectra as *Y*(*f*) functions. For a pixel-by-pixel analysis of the zero-temporal-mean dynamic images, the signal-time curve at each pixel was Fourier transformed into a complex-valued spectrum, and they were *Y*(*f*). The spectra of the FastICA segmentation results showing either good respiratory or cardiac pulsations were selected as the reference spectra, *X*(*f*). By using (3), the complex *H*(*f*) value were calculated. The averaged values of |*H*(*f*)| and *ϕ*(*f*) within a selected frequency band was calculated to represent the strength and temporal delay information of the pulsation at this frequency band, respectively.

## 3. Results

**Figure 1:**
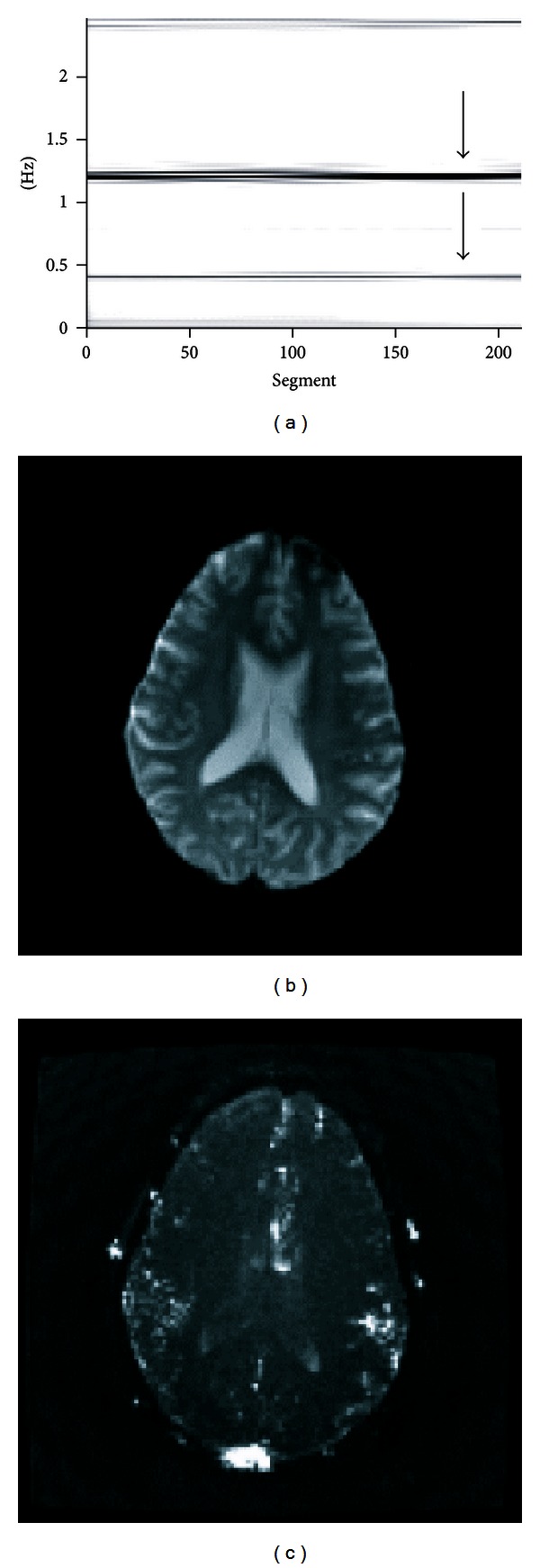
Analysis of a dataset acquired from a slice through the third ventricle. The 180th to the 479th dynamic images had stable respiratory and cardiac pulsations (pointed by arrows) as shown in the periodogram (a). The first image of the 512 dynamic images is used to display anatomy (b). Temporal-standard-deviation image illustrates the flow-related enhancement caused by oscillatory flows (c).

**Figure 2:**
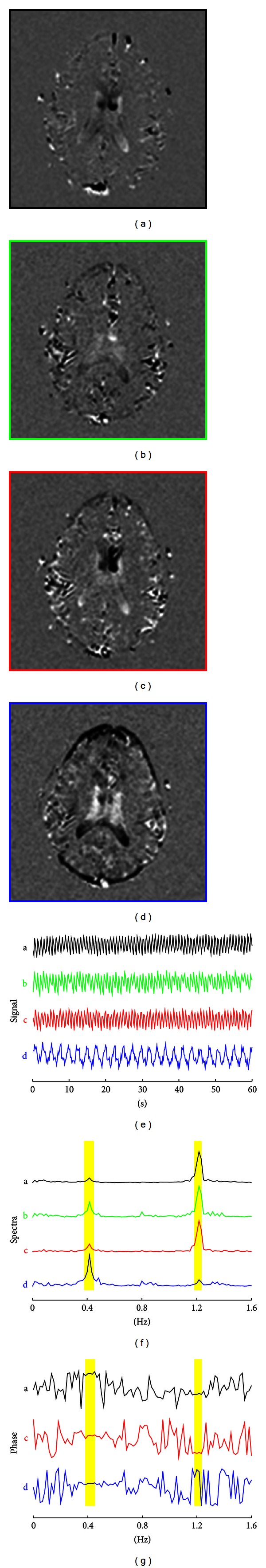
FastICA segmentation results for the dynamic images are displayed in Figure 1, which include the following: four output images with their frames displayed in different colors (a–d), corresponding signal-time curves (e), and corresponding amplitude spectrum (f). The phase, *ϕ*(*f*), calculated by using transfer function analysis and the complex-valued spectrum of the green signal-time curve as a reference function are illustrated in (g). The respiratory and cardiac frequency bands are marked by yellow shaded areas. Note that constant phases are observable at the respiratory (black, red, and blue curves) and cardiac pulsations (black and red curves).

**Figure 3:**
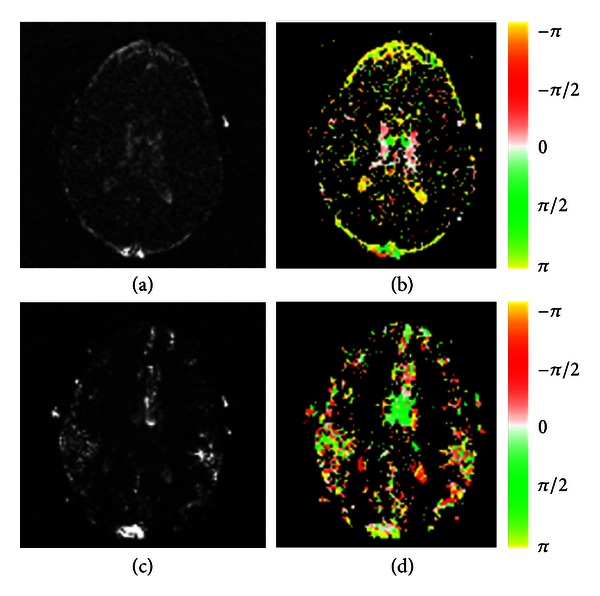
The amplitude (first column) and phase (second column) images are calculated by using the transfer function analysis for the respiratory (first row) and cardiac (second row) frequency bands. A pixel with a negative value (displayed in red color) has a signal-time curve leading the reference function. A pixel with a positive value (displayed in green color) indicates its signal-time curve is lagging the reference signal. The white color implies no or very small delays, and yellow color implies either a leading or a delay of *T*/2.

The postprocessing procedure for a dataset acquired from a slice through the ventricle is demonstrated in Figures 1–3. The FastICA calculation results for 300 zero-temporal-mean dynamic images are shown in Figure 2, including four output images (a–d) and corresponding signal-time curves (e). Spatiotemporal patterns for the respiratory and cardiac pulsations are well demonstrated in these results.  Figure 2(f) displays the corresponding amplitude spectra. Peaks were found at the respiratory (0.42 Hz) and the first harmonic (1.22 Hz) of cardiac frequency bands, which are marked by yellow shaded areas.  Figure 2(g) plots the phase, *ϕ*(*f*), of the transfer function, *H*(*f*), obtained from complex divisions using the first (black), third (red), and fourth (blue) spectra of the FastICA segmentation results, divided by the second (green) spectrum of the FastICA segmentation results. Near constant phases were found at the marked respiratory and cardiac frequency bands, indicating the existence of phase delay at these frequency bands.

The |*H*(*f*)| and *ϕ*(*f*) images at frequency bands that correspond to respiratory and cardiac pulsations are displayed in Figure 3. In the transfer function calculation, the green signal-time curve displayed in Figure 2(e) was used as *x*(*t*). A 180-degree phase delay or inverted amplitude is observed at pixels with red versus green colors or pixels with yellow versus white colors. 

**Figure 4:**
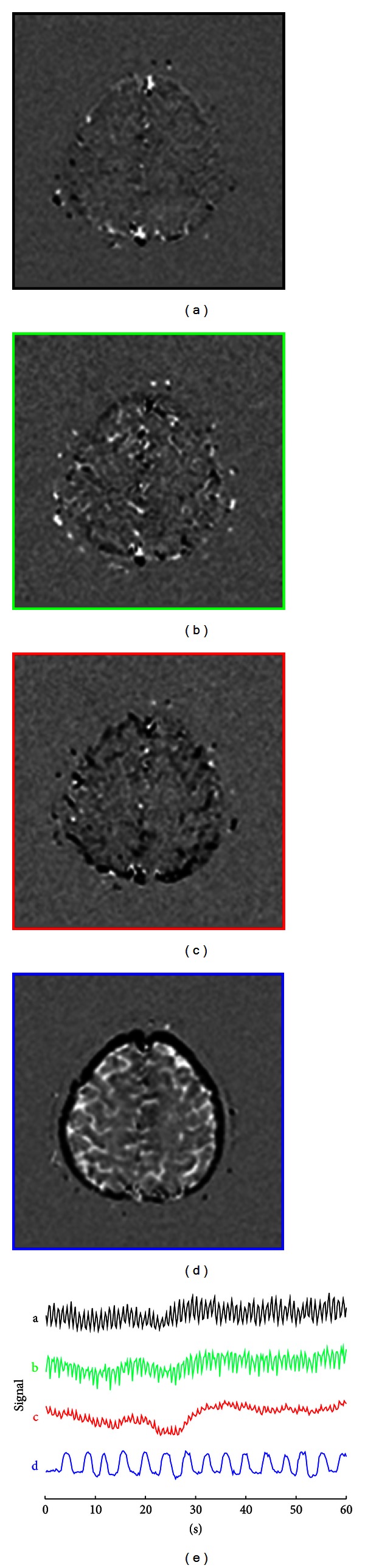
FastICA segmentation results for a slice location above the third ventricle, including four output images (a–d), and corresponding signal-time curves (e).

**Figure 5:**
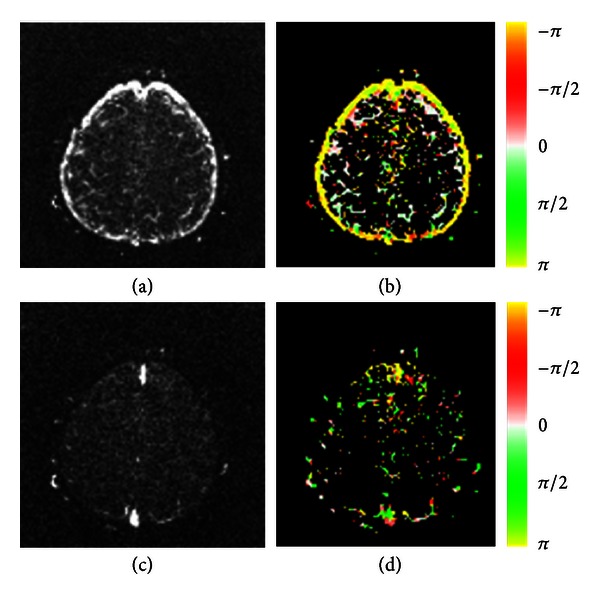
Transfer function analysis of the dynamic images shown in Figure 4. The amplitude (first column) and phase (second column) images were calculated by using the transfer function analysis for the respiratory (first row) and cardiac (second row) frequency bands. The color coding is the same as that in Figure 3.

The FastICA segmentation results for a slice above the ventricle are shown in Figure 4. Pixels at the inner and outer sides of the brain surface are displayed in white and black colors in Figure 4(d). This phenomenon indicates that the signal-time curves at these two regions had either a 180-degree phase delay or inverted amplitudes. The |*H*(*f*)| and *ϕ*(*f*) images at frequency bands that correspond to respiratory and cardiac pulsations are displayed in Figure 5. Again, the 180-degree phase delay or inverted amplitude is observed at pixels with yellow versus white colors in Figure 5(b).

## 4. Discussion

 We analyzed the respiratory and cardiac pulsations in human brain using the transfer function analysis. By using the reference spectra produced from the FastICA segmentation results, the magnitude and phase images at the respiratory and cardiac frequency bands were calculated. The magnitude represented the strength of these pulsations observed at different brain locations. The phase was related to the temporal delay in the periodical waveforms compared with the reference signals. The respiratory and cardiac pulsations were analyzed separately. The assumption of spatial independency used in the IC analysis was not needed in the transfer function analysis. Furthermore, the phases, as well as the temporal delays, were calculated as continuous numbers as shown in Figures 3 and 5. On the contrary, only black and white colors were used on the output of FastICA segmentation results as shown in Figures 2 and 4. The temporal delay information is limited to either 0 degree or 180 degrees in the IC analysis.

It is not surprising to know that the cardiac pulsation is observed at intracranial vessels as illustrated in Figures 3(c), 3(d), 5(c), and 5(d). In the ventricle, both respiratory and cardiac pulsations were observed as shown in Figure 3. We postulate that the respiratory pulsation is propagated through venous blood and is originated by far-away intrathoracic pressure changes, secondary to respiration. Meanwhile, the cardiac pulsation might be caused by a nearby cardiac pulsation at either choroid plexus or brain matter.

Because the CSF space is part of a closed system, oscillatory CSF flow that went into one direction must be compensated by the same amount of oscillatory CSF flow that went to the opposite direction. It is likely that the flow pattern of the cortical CSF at the brain surface is companied by the opposite CSF flow pattern in the brain as shown in Figures 4(d) and 5(b). 

The CSF is produced in the choroid plexus and circulates through the ventricular system, subsequently draining into the CSF space on brain surface, where it is resorbed from the superior sagittal and other sinuses before returning to the systemic circulation [2, 24, 25]. The intracranial pressure or CSF space will increase when there is overproduction of CSF, obstruction of CSF circulation, or brain atrophy. These three conditions all result in ventricular dilatation although their intracranial pressures are different. Clinically, the former two conditions are correctable while brain atrophy is irreversible. In addition to the CSF bulk flow mentioned previously, animal models have shown that either an increase or a decrease of cardiac pulsation in ventricular CSF causes ventricular dilatation [26]. It is reasonable to propose that CSF bulk flow and CSF pulsation are related to each other, and an imbalance of these flow dynamics may cause CSF flow obstruction with resulting ventricular dilatation. For clinical applications, the spatiotemporal patterns of respiratory and cardiac pulsations in patients with ventricular dilatation or hydrocephalus might provide information on the circulation of CSF.

There are two drawbacks in the transfer function analysis. The first drawback is that the phase is limited between ±*π*. As a consequence, the temporal phase delay, *τ*_*p*_(*f*), is limited between ±*T*/2, where *T* = 1/*f*. This condition may cause ambiguity in clinical applications [18], because an inverted signal cannot be distinguished from a signal with a phase delay of *T*/2 for a periodical waveform. The inverted amplitude or 180-degree phase delay phenomenon was observed on Figures 3(b), 3(d), and 5(b).

The second drawback is that the second harmonic of cardiac pulsation is limited by the Nyquist sampling rate of the dynamic images. In this study, the Nyquist sampling rate is calculated as 1/*T*_*R*_/2 = 2.5 Hz [27]. For the second harmonic of the cardiac pulsation to be observable on the dynamic images, the fastest heart rate is limited to 1.25 Hz, which corresponds to 75 heart beats per minute. If a subject's heart rate exceeds this limit, the second harmonic of the cardiac pulsation cannot be found in the dynamic images, and aliasing effect may interfere with respiratory pulsation.

## 5. Conclusion

This paper presented a protocol for analyzing the spatiotemporal patterns of respiratory and cardiac pulsations in human. The respiratory and cardiac pulsations can be recorded as flow-related enhancement on dynamic MR images. The segmentation results of IC analysis can be used to provide a reference function for the transfer function analysis. In the transfer function analysis, we found the following: (1) a good delineation of temporal delay of these pulsations can be achieved; (2) respiratory pulsation exists in the ventricular and cortical CSF; (3) cardiac pulsation exists in the ventricular CSF and intracranial vessels; and (4) a 180-degree phase delay or inverted amplitude is observed on phase images.
